# Inferior Pole of the Patella Fracture Fixed by Percutaneous Approach: A Case Report

**DOI:** 10.7759/cureus.66013

**Published:** 2024-08-02

**Authors:** Abhishek Nair, Ashwin Deshmukh, Swaroop Solunke, Shubhankar Chopra, Archit Gupta

**Affiliations:** 1 Orthopaedics, Dr. D. Y. Patil Medical College, Hospital and Research Centre, Dr. D. Y. Patil Vidyapeeth (Deemed to be University), Pune, IND

**Keywords:** inferior pole of the patella, elderly trauma, novel surgical technique, percutaneous procedure, patella fracture

## Abstract

Fractures of the inferior pole of the patella are rare but challenging orthopedic injuries. This case study presents the management of such a fracture using a percutaneous approach. A 70-year-old female patient presented with significant knee pain and swelling following a fall. Radiographic examination revealed a displaced fracture of the inferior pole of the patella along with an ipsilateral tibial plateau fracture. Surgical intervention was deemed necessary due to the extent of displacement and the potential for compromised knee function. A percutaneous technique was employed for fracture reduction and fixation using cannulated screws under fluoroscopic guidance. Postoperative rehabilitation focused on early mobilization and strengthening exercises. At a six-week follow-up, the patient demonstrated satisfactory clinical outcomes with restoration of knee function and minimal residual symptoms. This case highlights the efficacy of percutaneous fixation in managing inferior pole patellar fractures, offering a minimally invasive approach with favorable functional outcomes.

## Introduction

Epidemiologically, patellar fractures account for approximately 1% of all fractures, with an age predominance in the middle age group with a demographic of 2:1 among males and females, respectively. The biomechanics of the patella is that it increases power and aids the extensor mechanism by 40% by pushing it away from the center of rotation [1].

Inferior pole patella fracture is a unique fracture that occurs in the distal one-fourth of the patella at the attachment of the patellar tendon, comprising cancellous bone, no articular surface, and not participating in the configuration of the patellofemoral joint. Patella fractures are classified on the basis of fracture pattern and degree of articular disruption [1]. Schatzker grading was used to classify the tibial fracture [2].

Here, we present a case of an inferior pole of the patella fracture managed through an unconventional closed reduction internal fixation approach.

## Case presentation

A 70-year-old female presented to our hospital with a chief complaint of left knee pain persisting for five days following a recent fall. There was no concurrent history of head injuries. She has a documented history of hypertension over the past three years, managed effectively with medication, and has no other notable medical, familial, or past medical history.

Clinical examination revealed no open wounds but demonstrated swelling and tenderness localized to the left knee. The patient exhibited a restricted range of motion due to pain, notably an inability to fully extend the knee.

Preoperative workup

**Figure 1:**
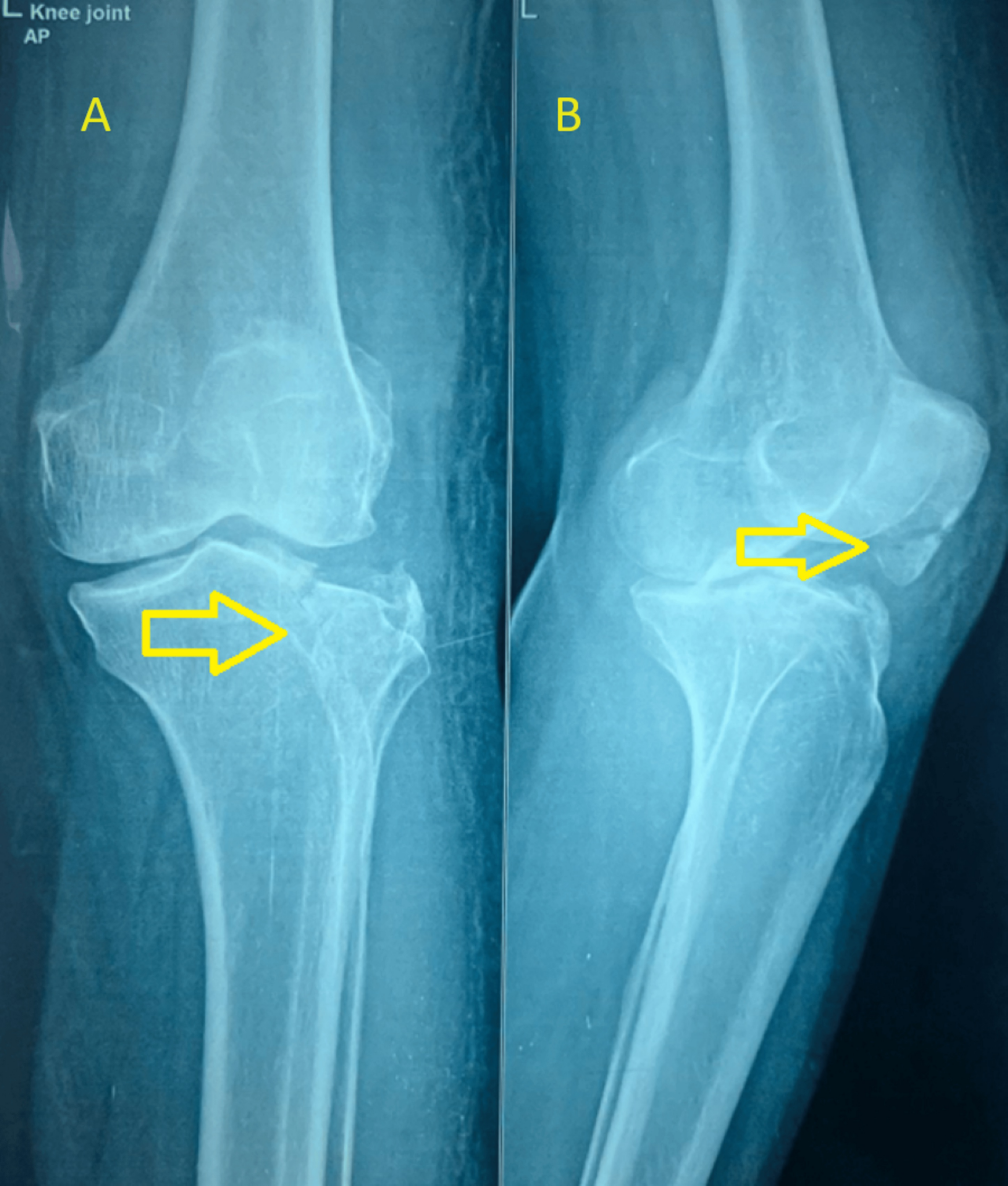
Plain radiograph of the left knee in both (A) anteroposterior and (B) lateral views The radiograph shows a fracture in the inferior pole of the patella, along with a fracture in the tibial plateau. Fractures are marked with yellow arrows

A radiograph taken after admission showed an inferior pole of the patella fracture along with a lateral tibial plateau fracture, which was classified as Schatzker type 3A (Figure 1).

**Figure 2:**
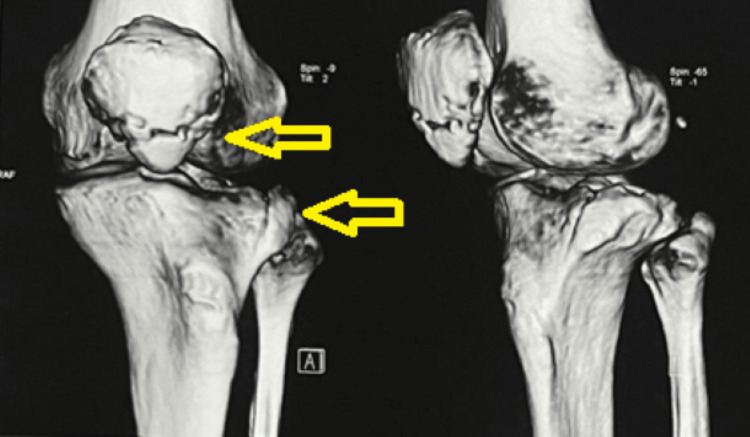
The 3D CT scan of the patella and tibia showing fractures marked with yellow arrows

**Figure 3:**
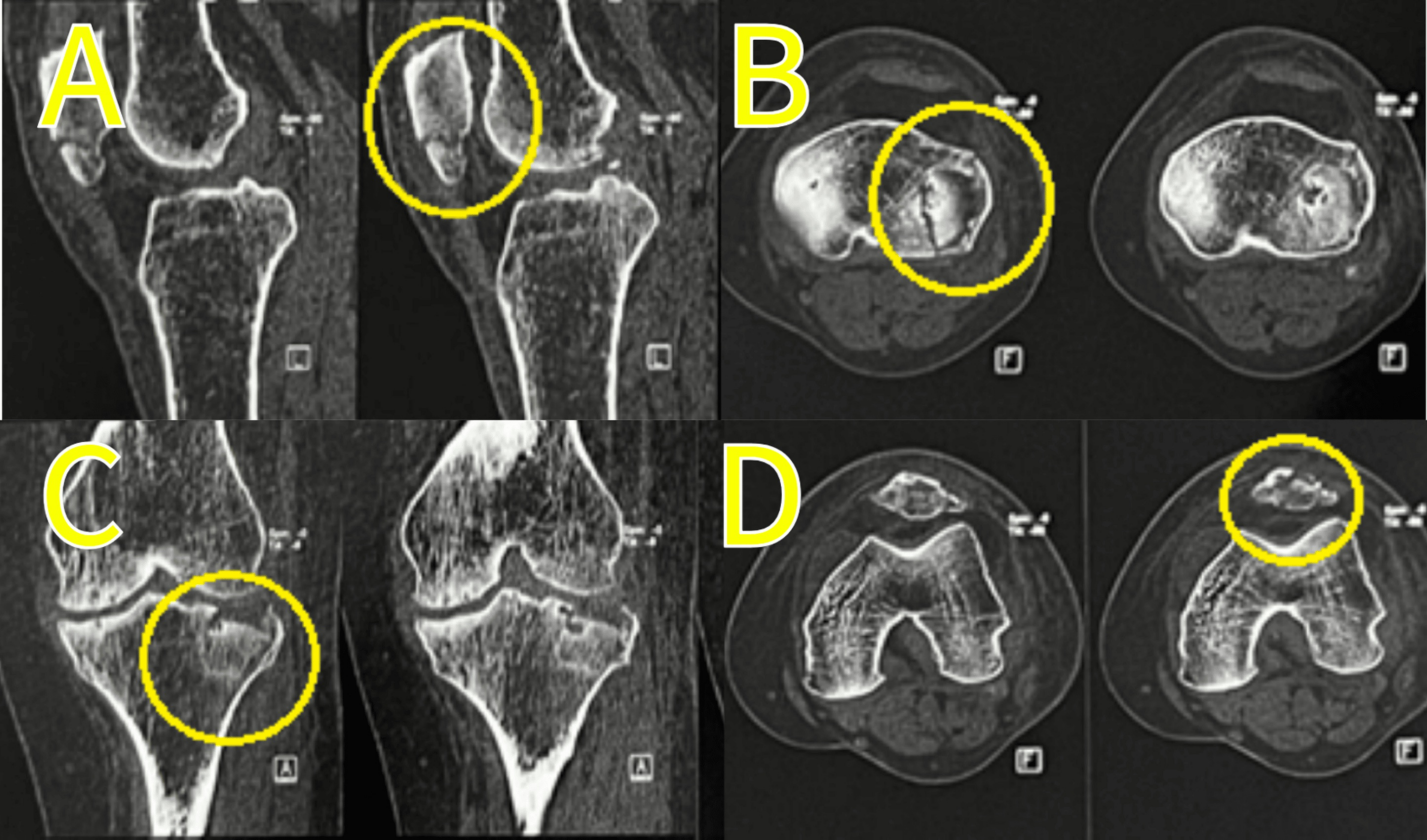
CT scan of the patella and proximal tibia (A) Sagittal view showing a lower pole of the patella fracture; (B) axial view of the proximal tibia showing a Schatzker type 3A fracture; (C) coronal view showing a split-depressed fracture of the proximal tibia; (D) axial view of the lower end of the patella showing a fracture

A CT scan was done and is shown in Figures 2, 3.

Intraoperative observation

After a thorough preoperative evaluation and pre-anesthesia checkup (PAC), the patient underwent surgery under spinal anesthesia. The fractured tibia was approached first. The patient was placed in a supine position with the knee flexed to 30°, and the tourniquet was inflated. A proximal lateral tibial plateau fracture was approached from the anterolateral aspect for ORIF (open reduction internal fixation), and buttress plating was done.

**Figure 4:**
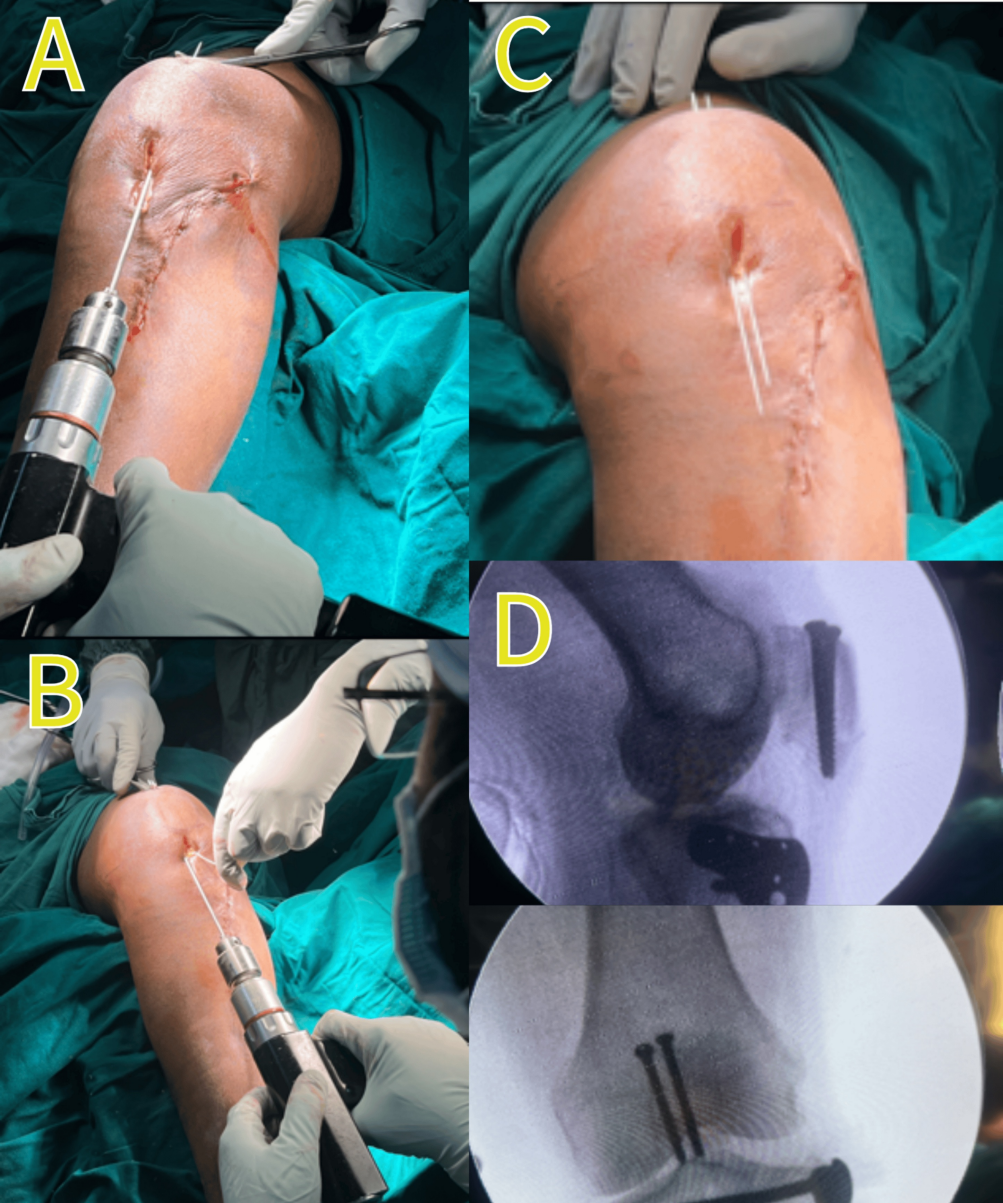
Intraoperative images (A) Passing the first guide wire percutaneously; (B) passing the second guide wire parallel to the first one; (C) the view after passing both guide wires; (D) intraoperative image intensifier images showing lateral and anteroposterior (AP) views of the patella

The superior pole of the patella was palpated and then secured in position using a Weber clamp. Subsequently, we passed two guide wires on either side of the clamp reduction to secure the patella in its place for closed reduction internal fixation. Concurrently, the cannulated compression screw (CC screw) was passed (Figure 4). The anterolateral approach used for the tibial plateau fracture made tension band wiring an unfavorable operative approach due to the potential for additional skin damage that could impede healing.

Postoperative rehabilitation

**Figure 5:**
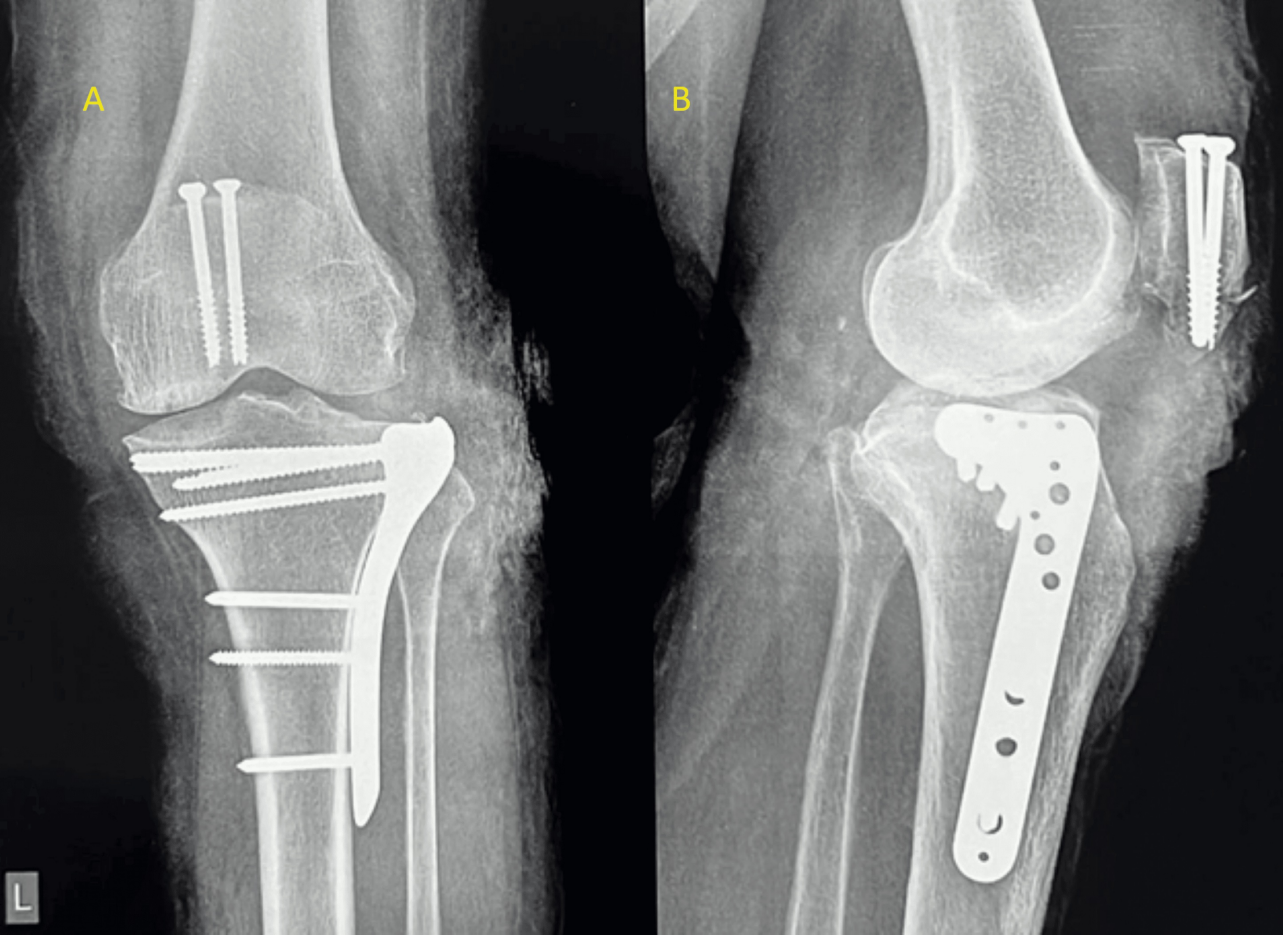
Postoperative radiograph of the left knee Radiograph showing a cannulated compression screw fixation for the inferior pole of the patella and buttress plating for the tibial plateau fracture (A) Anteroposterior view; (B) lateral view

A long knee brace was applied immediately after surgery. On the second postop day, debulking of the dressing was done, following which the patient was asked to commence a passive range of movement. The postoperative radiograph is shown in Figure 5.

## Discussion

There is no fixed consensus for a definite approach to an inferior pole of the patella fracture. The modality of treatment is based on patient-specific parameters such as age, type, and additional fractures. The traditional approach to treating a patella fracture involves ORIF using the mid-axial longitudinal approach. Preferred operative modalities for this procedure include tension band constructs utilizing k-wires, cannulated screws, and sutures; the use of plates and screws, such as mini-fragment plates or mesh plates; and cerclage wiring, with or without primary fixation.

Tension band wiring, although it provides a good postoperative recovery and functional exercise prognosis, is not vastly effective in the immobilization of a lower pole patella fracture due to its tendency to loosen after surgery, thus causing immobilization failure [3]. Cerclage wiring has not been exceedingly successful in immobilization, and there have been reports of fracture block separation and rotational displacement that have occurred in a matter of a few months following the procedure [4].

Postoperative rehabilitation notably depends on the surgical technique used, the fixation device, and early encouragement to start range of motion (ROM) training.

## Conclusions

In this case study, we explored the successful management of an inferior pole patella fracture in a 70-year-old female patient through a percutaneous closed reduction internal fixation approach. This method was chosen due to the specific nature of the injury and the need to minimize additional skin damage, which was critical given the concurrent tibial plateau fracture treated via an anterolateral approach.

Postoperative outcomes demonstrated minimal recovery time and effective rehabilitation, highlighting the efficacy of this approach for similar fractures. The patient's recovery, facilitated by early mobilization and appropriate bracing, underscores the importance of tailored treatment plans in orthopedic surgery. This case reinforces that while traditional methods, such as tension band wiring, have their place, alternative techniques can offer significant advantages in specific clinical scenarios, particularly in managing complex fractures with concurrent injuries.
